# Books and Magazines

**Published:** 1903-02

**Authors:** 


Annual Report of the Smithsonian Institution.—One of the functions of the Smithsonian Institution, at Washington, is the diffusion of knowledge in language "understanded of the people;" so that, while most of its works are intended primarily for the specialist, there is an exception made by the secretary in publishing an appendix to the report of the board of regents, which is in fact an annual summary of the most interesting events of the scientific year, prepared for that large body of the public which does not care for professional memoirs, but has a general interest in such matters.

This popular volume for 1901 is before us. It contains fifty articles, many of them illustrated, nearly all prepared by masters of the respective subjects, telling in clear and interesting language of the latest progress in all the principal branches of knowledge.

A short sketch of the history and the work of the Smithsonian Institution, begins with a paragraph from President Roosevelt's first message to Congress, in which he calls attention to the institution's functions and its present needs. The paper further states that the Smithsonian Institution, which is composed of the president and his cabinet, and the vice-president and chief justices of United States, has a remarkable organization for the administration of funds for the promotion of science. Its activities could be still further increased if it had greater means at its absolute disposal; while those who are thinking of giving for some special scientific object may yet find the regents, on account of the peculiarly disinterested position they hold, the best counsellors in suggesting the channel into which gifts for public purposes might be directed, even should they not see their way clear to accepting such donations for the institution itself.

"Bodies Smaller than Atoms" is the title of an interesting paper, and as we read "The Laws of Nature," "The Greatest Flying Creature," and "The Fire Walk Ceremony at Tahiti," we are reminded of the wide range of subjects included in the report. Wireless telegraphy, transatlantic telephoning, and the telephonograph are discussed by experts in electrical progress. Attention ought also to be called to papers on utilization of the sun's energy, the Bogosloff volcanoes of Alaska, forest destruction, irrigation, the children's room at the Smithsonian, the submarine boat, a new African animal, pictures by prehistoric cave-dwellers in France, automobile races, the terrible lizards that once lived in America, and Mr. Thompson-Seton's paper on the National Zoölogical Park at Washington.

The whole volume has been called "the best popular scientific annual published in the world."

The Smithsonian reports are distributed by the institution to libraries throughout the world; may be had by purchase at cost from the superintendent of documents, Washington City, *and may also generally be obtained free of charge from the applicant's member of Congress.*

---

It is a peculiar fact that the letters and others writings of DeQuincey, Carlyle, Darwin, Huxley and Browning, liberal as they are with references to the continued ill-health of those great writers, have not before this suggested to the medical profession an opportunity for research into the causal factors of those physical conditions. That the opportunity has not until now been recognized in its proper light is evidenced by the hitherto total absence of any work dealing with this subject. Dr. George M. Gould's Biographic Clinics (P. Blakiston's Son & Co., Philadelphia), which is devoted to this neglected subject should, therefore, prove a most unique and valuable contribution to biographical and medical literature. The work is announced for publication in December.

Mr. Gould has gathered from the biographies, writings and letters of the five named men every reference to their ill-health. Each endured, as is well known, a life of suffering which made almost every day a torment and by which their work and worth as an asset of the nation and civilization was conditioned and often rendered morbid. The cause of their affliction was an utter mystery to their physicians. No explanation explained, and no cure cured. Dr. Gould has gone into the "why" of this very thoroughly and the conclusion reached by him, from logic and from a careful summary of the clinical symptoms, is that each of the writers suffered from eyestrain, and that scientific correction of their ametropia would have transformed their lives of misery into lives of happiness. A history of the discovery of astigmatism and eyestrain, with a discussion of its indications and responsibilities, completes the work. It is interestingly written, and will undoubtedly meet with a ready sale among medical men and those interested in the works and lives of the quintette of great writers.

